# Tutorial for using SliceOmatic to calculate thigh area and composition from computed tomography images from older adults

**DOI:** 10.1371/journal.pone.0204529

**Published:** 2018-10-02

**Authors:** Richard A. Dennis, Douglas E. Long, Reid D. Landes, Kalpana P. Padala, Prasad R. Padala, Kimberly K. Garner, James N. Wise, Charlotte A. Peterson, Dennis H. Sullivan

**Affiliations:** 1 Geriatric Research, Education and Clinical Center, Central Arkansas Veterans Healthcare System, North Little Rock, Arkansas, United States of America; 2 Donald W Reynolds Department of Geriatrics, University of Arkansas for Medical Sciences, Little Rock, Arkansas, United States of America; 3 College of Health Sciences and Center for Muscle Biology, University of Kentucky, Lexington, Kentucky, United States of America; 4 Department of Biostatistics, University of Arkansas for Medical Sciences, Little Rock, Arkansas, United States of America; 5 Department of Psychiatry, University of Arkansas for Medical Sciences, Little Rock, Arkansas, United States of America; 6 College of Medicine, Department of Radiology, University of Arkansas for Medical Sciences, Little Rock, Arkansas, United States of America; Charles P. Darby Children's Research Institute, UNITED STATES

## Abstract

**Objective:**

Area of muscle, fat, and bone is often measured in thigh CT scans when tissue composition is a key outcome. SliceOmatic software is commonly referenced for such analysis but published methods may be insufficient for new users. Thus, a quick start guide to calculating thigh composition using SliceOmatic has been developed.

**Methods:**

CT images of the thigh were collected from older (69 ± 4 yrs, N = 24) adults before and after 12-weeks of resistance training. SliceOmatic was used to segment images into seven density regions encompassing fat, muscle, and bone from -190 to +2000 Hounsfield Units [HU]. The relative contributions to thigh area and the effects of tissue density overlap for skin and marrow with muscle and fat were determined.

**Results:**

The largest contributors to the thigh were normal fat (-190 to -30 HU, 29.1 ± 7.4%) and muscle (35 to 100 HU, 48.9 ± 8.2%) while the smallest were high density (101 to 150 HU, 0.79 ± 0.50%) and very high density muscle (151 to 200 HU, 0.07 ± 0.02%). Training significantly (P<0.05) increased area for muscle in the very low (-29 to -1 HU, 5.5 ± 7.9%), low (0 to 34 HU, 9.6 ± 16.8%), normal (35 to 100 HU, 4.2 ± 7.9%), and high (100 to 150 HU, 70.9 ± 80.6%) density ranges for muscle. Normal fat, very high density muscle and bone did not change (P>0.05). Contributions to area were altered by ~1% or less and the results of training were not affected by accounting for skin and marrow.

**Conclusions:**

When using SliceOmatic to calculate thigh composition, accounting for skin and marrow may not be necessary. We recommend defining muscle as -29 to +200 HU but that smaller ranges (e.g. low density muscle, 0 to 34 HU) can easily be examined for relationships with the health condition and intervention of interest.

**Trial registration:**

Clinicaltrials.gov NCT02261961

## Introduction

Skeletal muscle content of the thigh is commonly measured in studies of health maintenance, aging, and disease due to its essential roles in functional ability and metabolism [1, 2]. Thigh composition is also of great interest since the relative contributions of muscle, fat, and bone to thigh area are health indicators or determinants (e.g. normal versus frail, cachectic, sarcopenic, or sarcopenic obese) that can change significantly with disease progression or intervention (e.g. nutrition and exercise) [3, 4]. Computed tomography (CT) is a gold-standard in muscle imaging due to its high resolution and reliability [5, 6]. The CT scan provides a cross-sectional image of the thigh or other area of interest based on differential absorption of radiation due to tissue density. In the grey level image from the scan, darker areas are less dense and lighter areas are denser tissue. Density is quantified in Hounsfield units (HU) assigned to each image pixel relative to reference values of air (-1000 HU) and water (0 HU) and tissues possess a characteristic HU range (e.g. fat -190 to -30, muscle -29 to +150, and bone +152 to +1000) [7]. This combination of measured density and constant size (e.g. 0.25 mm^2^) for each image pixel enables calculation of area for each tissue of interest.

Calculation of tissue area from CT scans requires specialized image analysis software such as the commercially available graphics program SliceOmatic (Tomovision, Magog, Canada). This program has been considered easy to use and offers excellent technical support [8]. However, even though a Google Scholar search through 2017 indicates that over 500 articles contain the words “SliceOmatic + thigh + muscle”, we were unable to find instructions on using the program to calculate tissue areas of the thigh. Thus, the purpose of this publication is to provide a quick start guide to assist investigators in using SliceOmatic to measure tissue areas for thigh cross-sectional CT images. Two potentially important issues apparent in the literature will also be examined here. One issue is that studies have not consistently used the same density range to define muscle. Studies commonly use -29, 0, or 35 HU as the lower limit and 100, 150, or 200 HU as the upper limit depending upon whether the investigators are interested in “low density” or “normal density” muscle [5, 8–10]. The effect of choosing each of these cutoff points on the contributions of muscle to total thigh area will be presented. The second issue is that skin and bone marrow contain overlapping densities with muscle and fat but accounting for this overlap is typically not mentioned in study methods. Since SliceOmatic allows image editing to account for this density overlap, the effects of skin and marrow on the results for muscle and fat area will also be presented.

We are conducting a randomized placebo-controlled trial to determine if a nutritional supplement increases the gains in muscle size and strength obtained from 12-weeks of high intensity resistance training of the thighs by older (60–80 yrs) adults [11]. Older adults were chosen because of the serious consequences that aging has on muscle mass and composition [12]. Veterans of US military service were specifically chosen due to the funding source and the high prevalence of comorbidities that negatively influence muscle health in this population [13, 14]. The nutritional supplement being tested contains arginine, glutamine, and methylbutyrate (Muscle Armor, Abbott Laboratories) and was chosen due to evidence of benefit to human muscle health in the contexts of wasting disease and healthy exercise [15–18]. The study is ongoing so the supplement and placebo assignments remain blinded. However, the CT scans of the thigh obtained before and after training from these older adults were used here to complete the following objectives: 1) develop a tutorial for using SliceOmatic to calculate thigh composition and cross-sectional area, 2) determine the relative contributions to total thigh area of seven density ranges encompassing fat, muscle, and bone, and 3) determine whether accounting for density overlap with skin and marrow changes thigh composition the statistical inference for the effects of training on muscle, fat, and bone.

## Materials and methods

### Study participants

The work was performed with approval from the institutional review board (IRB) of the Central Arkansas Veterans Healthcare System (Protocol 608119, Clinicaltrials.gov registration: 10-10-2014, NCT02261961). All participants provided written and verbal informed consent. The participants were older (60–80 yrs) Veterans of U.S. military service who were non-smokers with a normal body mass index (BMI, 18.5–29.9 kg/m^2^) though an IRB-approved deviation allowed enrollment of one subject with a BMI of 30.7 kg/m^2^. The full eligibility criteria and protocol are available [11]. CT scans were obtained from participants before and after 12-weeks of high-intensity progressive resistance exercise training of the thigh muscles (leg press, knee extension and curl). Participants trained three times per week by completing three sets (60, 70, and 75% of 1-rep max) of 10 reps and a fourth set (80% of 1-rep max) to voluntary failure.

### CT scans

Scans of the mid-thigh of the dominant leg were obtained using the hospital computed tomography service. Participants rested in a supine position for 30 min prior to being positioned (optimally without inner thighs touching) in the scanner. Cross-sectional images were taken at the midpoint between the inguinal crease and proximal border of the patella along the femur based on a scout image. Images (2.5mm thick) were collected by standard algorithm at 120 kV, 100 mA, and 0.8 sec rotation time.

### Analysis software

**Fig 1:**
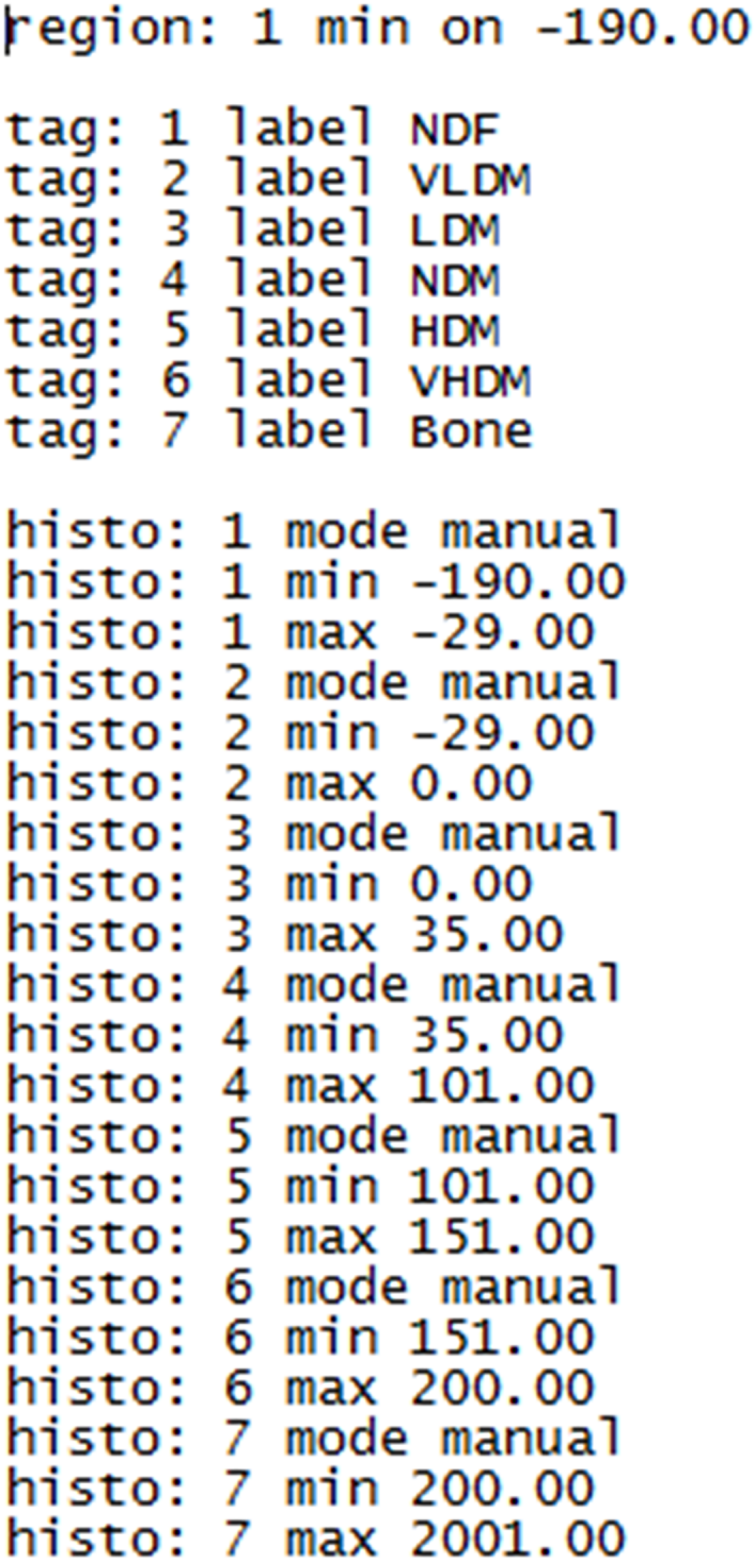
Step-by-step histogram method for segmentation of thigh tissue into fat, muscle, and bone and then calculating the cross-sectional area of each region. Prior to beginning, the file location and pathway of the CT scan images should be known. CT grey level images will often be in DICOM file format and may show the extension *.dcm though SliceOmatic can read image files in other formats too. SliceOmatic segments tissue into regions using a script file run by the program. The user must create this file by pasting the following script text into Notepad, or similar program, and saving as an ANSI text file whose name must end with the extension “.scp”. The script sets the color for each “tag” and the density ranges to be applied when the Region Growing (“region”) or Histogram Segmentation (“histo”) modes are used for segmentation. Script Text to be copied into Notepad File titled “Thigh Segmentation Tutorial.scp” is shown.

**Fig 2:**
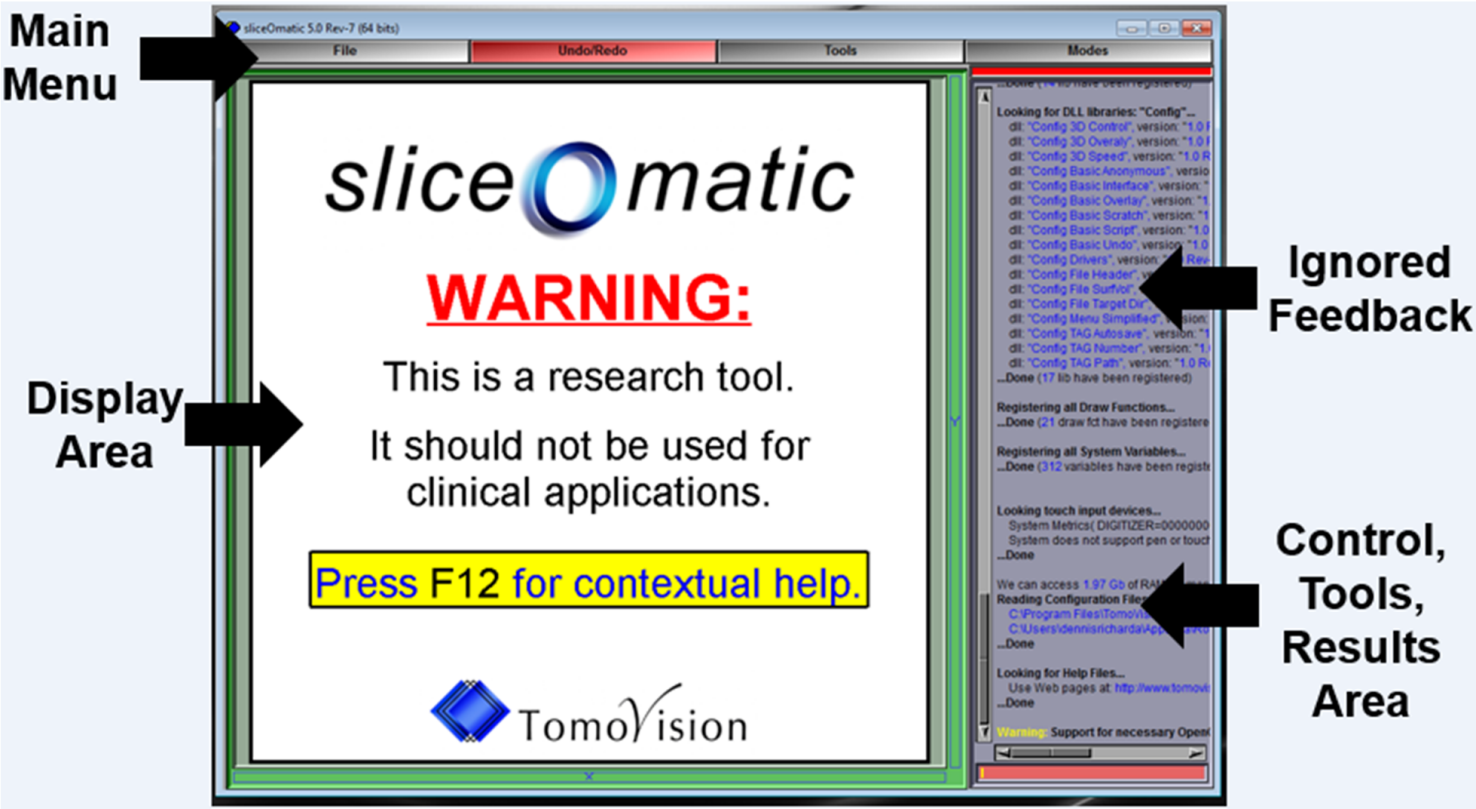
SliceOmatic is opened by double clicking the desktop icon or by following the Windows Start menu > All Programs > Tomovision > SliceOmatic. The open SliceOmatic window displays the Main Menu bar at the top and the Display Area on the left contains the word “WARNING”. The areas on the upper right contain mostly test feedback that can be ignored for this tutorial and the lower right contains the Control and Tools Areas which will later contain needed control buttons, sliders, and results. All images in Figs 2–12 have been reprinted from SliceOmatic v5.0 rev7 under a CC BY license, with permission from Tomovision, original copyright 2017.

**Fig 3:**
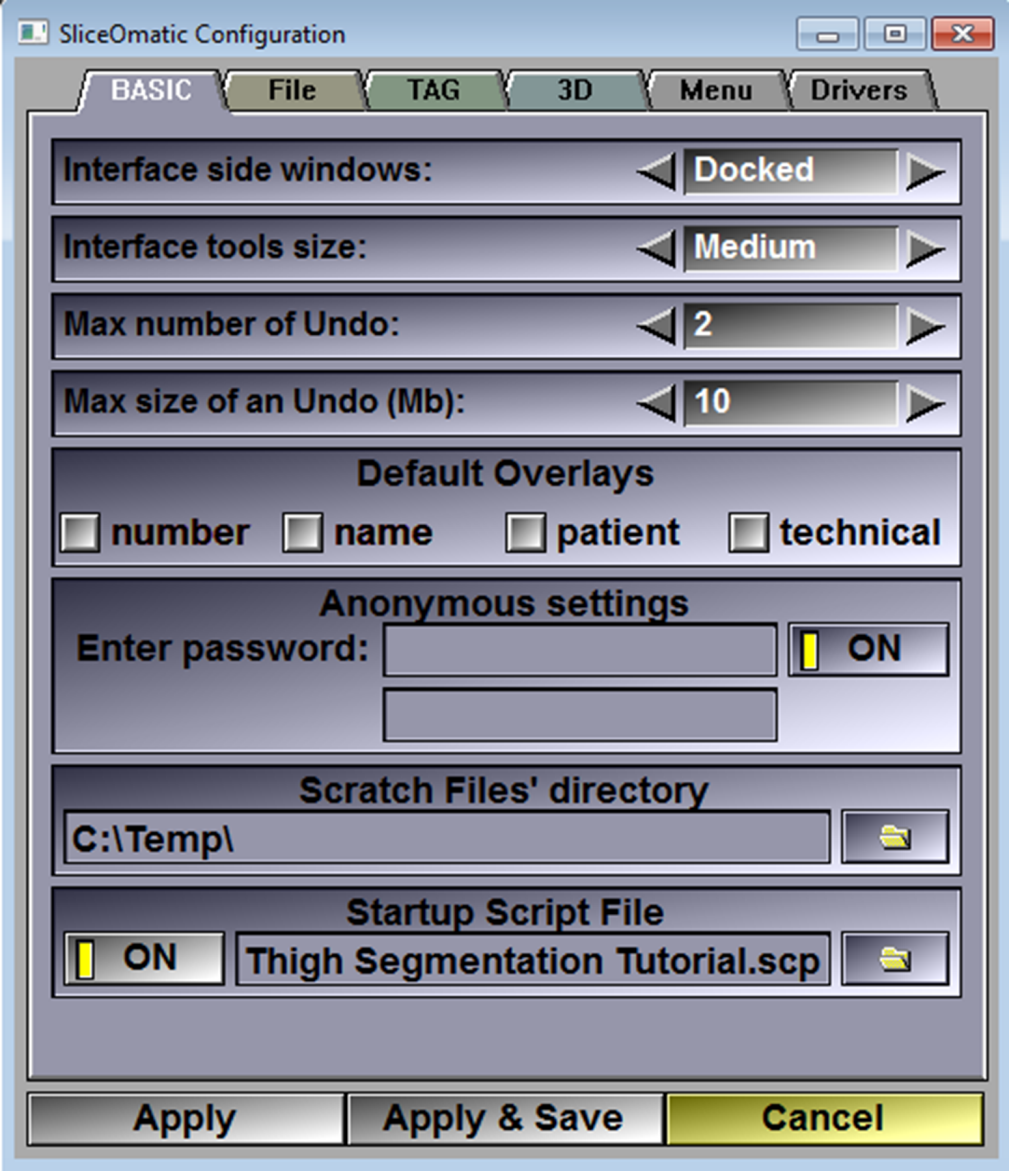
The script file (Thigh Segmentation Tutorial.scp) presets SliceOmatic analysis settings and is applied by dragging the file over into the Display Area. Alternatively, the script can be applied at startup every time SliceOmatic opens by following the Main Menu > File > Config > Startup Script File. Add the full path and script filename to the box, toggle OFF to ON, and apply and save.

**Fig 4:**
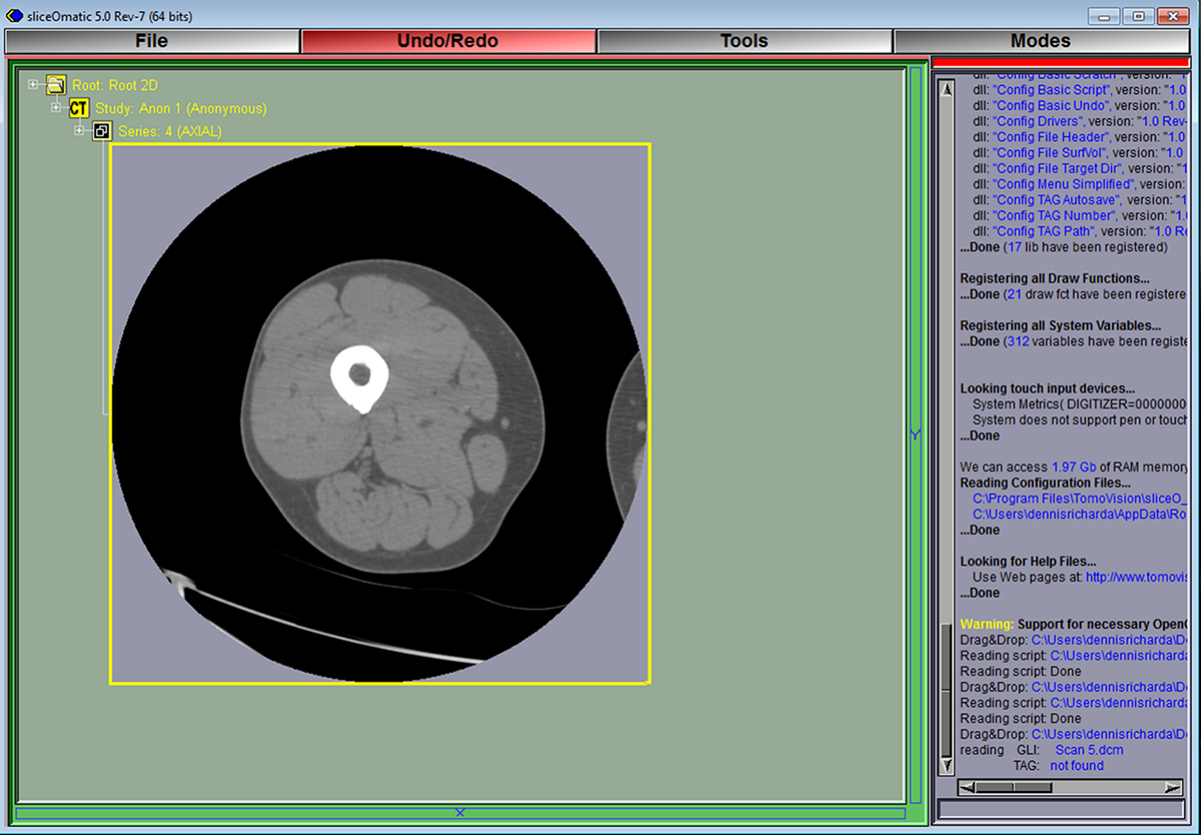
The CT grey level images or DICOM files should be available in a convenient known location such as a desktop folder. A CT scan is opened by dragging the image file into the Display Area. SliceOmatic can open multiple images at the same time but it is recommended that the user focuses on single images until familiar with the program and the analysis method.

**Fig 5:**
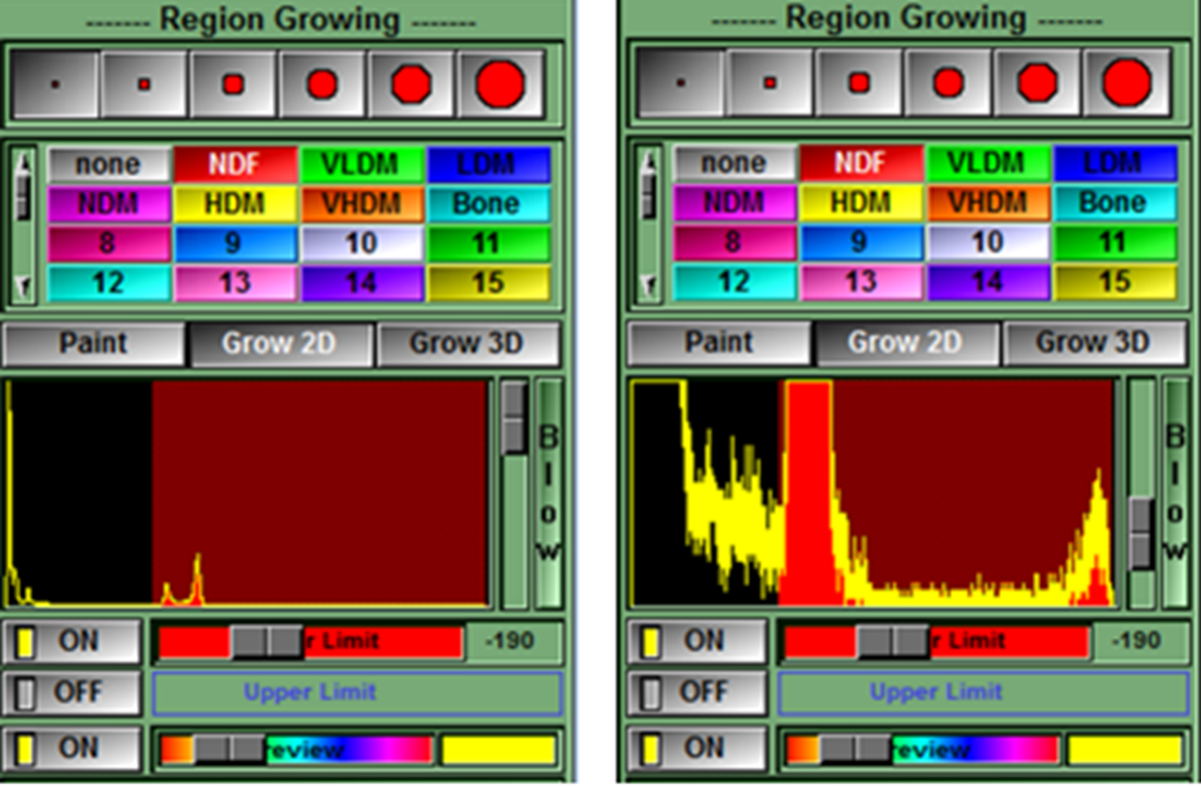
The thigh of interest is isolated by temporarily tagging all tissues with a density ≥ -190 HU (i.e. fat, muscle, and bone) using the region growing mode. Coloring will usually not escape the border of the thigh since it is mostly surrounded by areas with density less than the -190 HU limit (e.g. air at -1000 HU). This limit was set by the script text (region: 1 min on -190.00) shown in Fig 1. The Region Growing options are made available in the lower right of the Control Area by clicking in the Main Menu on Mode > Region Growing. The region growing options to be clicked are: smallest brush size (a bug in the program prevents a larger brush from being used), the temporary color (NDF i.e. red), and Grow 2D. The histogram shows that the peaks for fat (left peak) and muscle (right peak) are included in the red areas with a density lower limit of -190 HU (left image). The peak on the far left is air. The bone peak is to the far right in the histogram but too small to be seen without narrowing the range of the y-axis by moving the gray slider bar down (right image).

**Fig 6:**
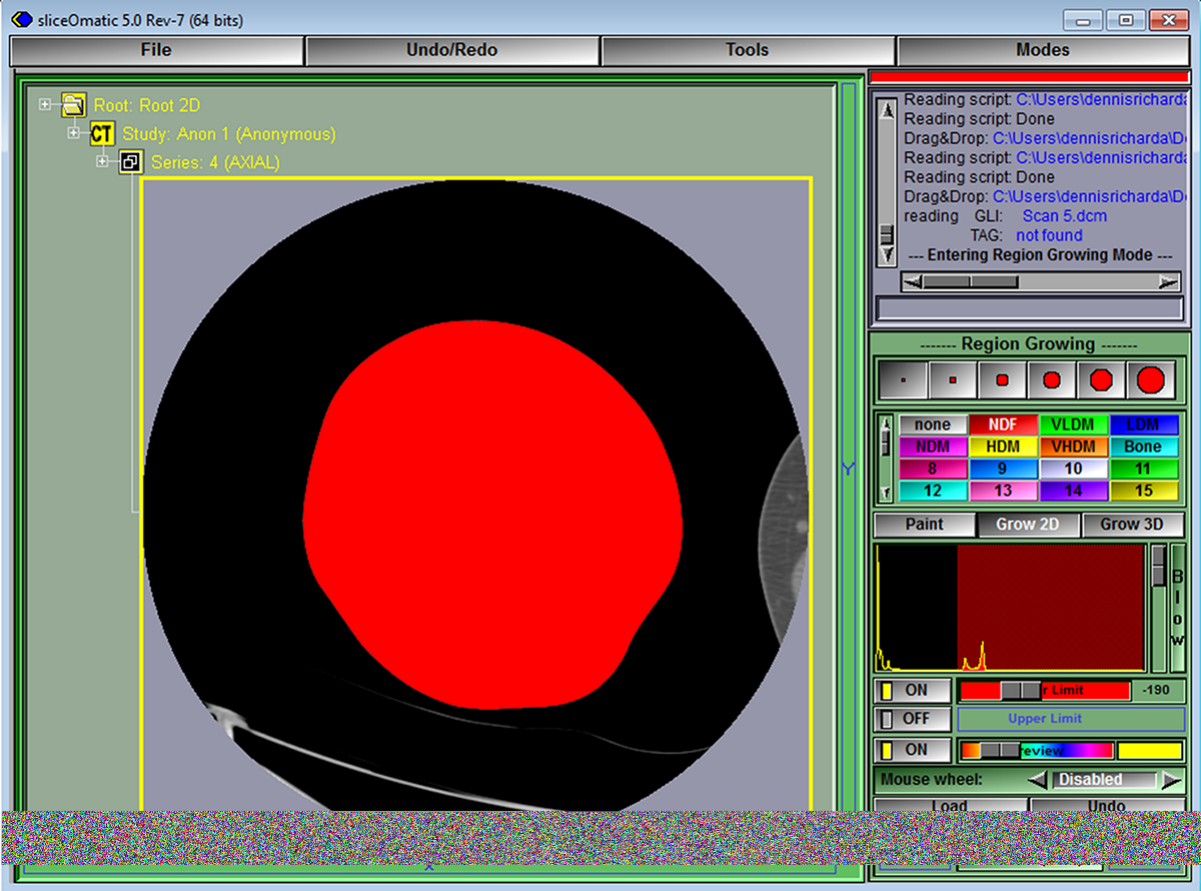
The thigh is then colored by clicking anywhere in its area. Sometimes areas touching the thigh (e.g. the opposite thigh or exam table) are also colored. Editing these inappropriately colored areas down to only the area of interest is presented later in this tutorial.

**Fig 7:**
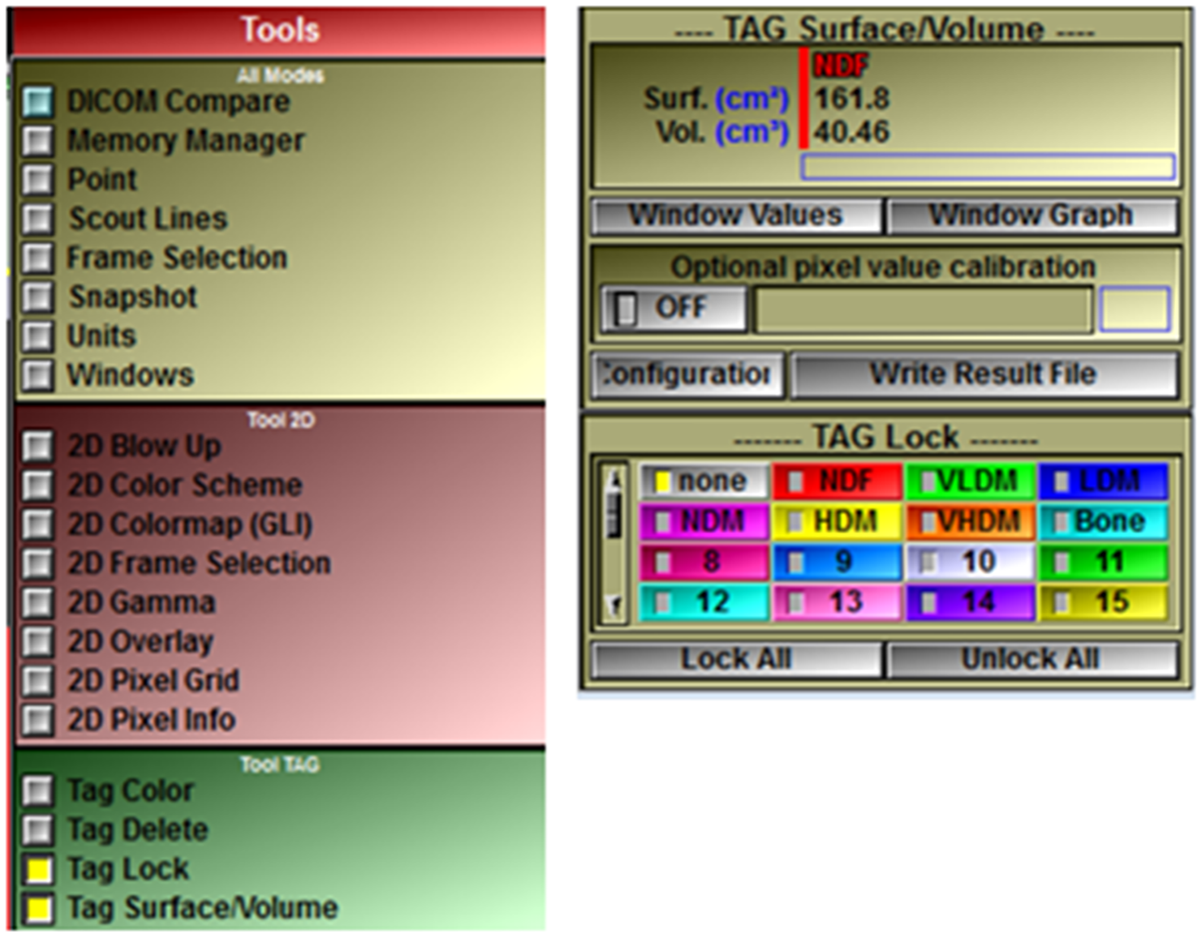
While the thigh is temporarily tagged red (NDF), further analysis is limited to this area by using Tag Lock to exclude untagged areas from segmentation. The size of tagged areas can also be shown. Click in the Main Menu on Tools > Tag Lock and Tag Surface/Volume to show these tools in the Control Area (note: the size of the SliceOmatic window likely needs to be maximized to see these tools and they may appear next to one another as shown here). Click Tag Lock “none” to exclude areas without a tag. The area of the red tag, i.e. total thigh area (161.8 cm^2^) is now shown and should be recorded or can be calculated later as the sum of all regions.

**Fig 8:**
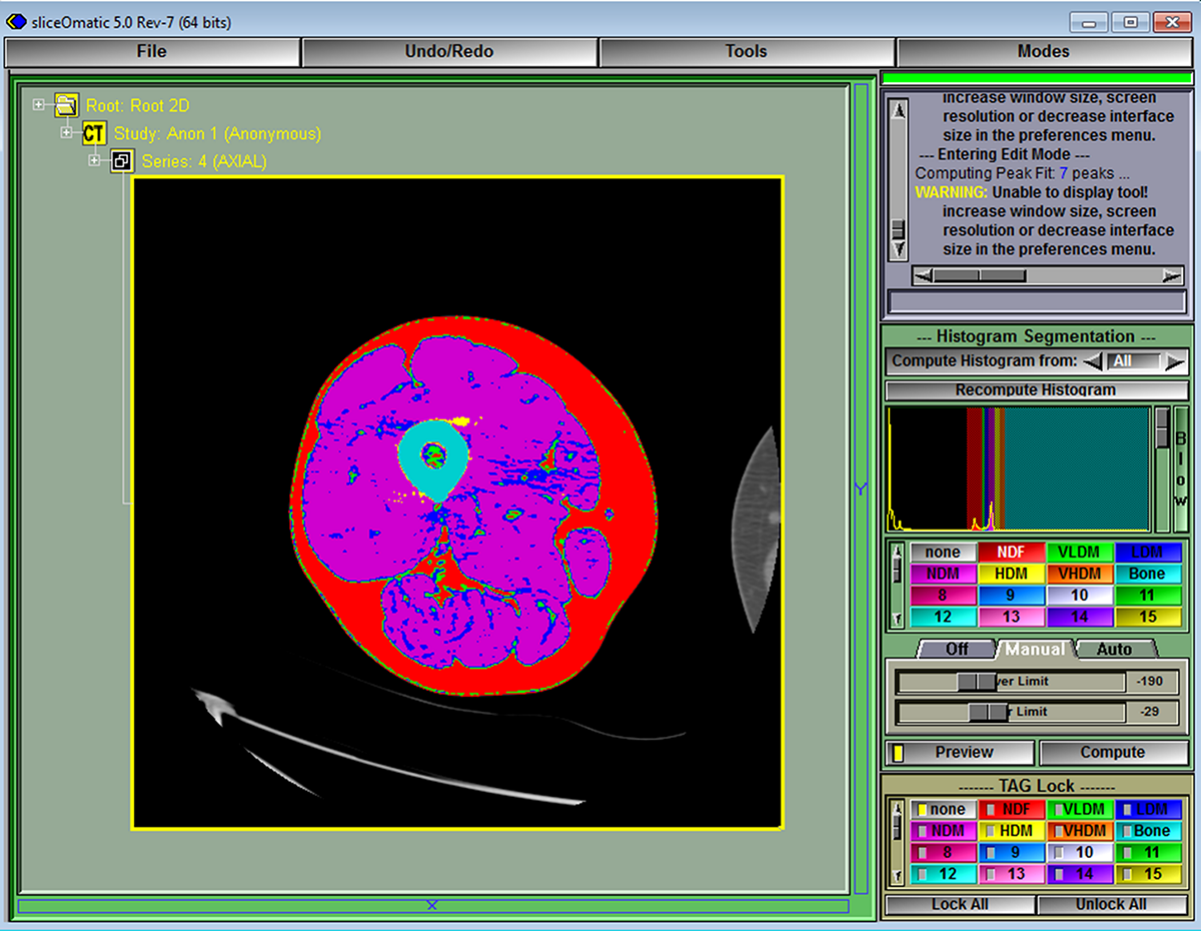
Segmentation into fat, muscle, and bone can now be performed based on the densities defined in Table 1 and set by the script in Fig 1. Click in the main menu on Modes > Histogram Segmentation to show the segmented thigh. If the opposite thigh also colors, then the tag lock was not set to “none”.

**Fig 9:**
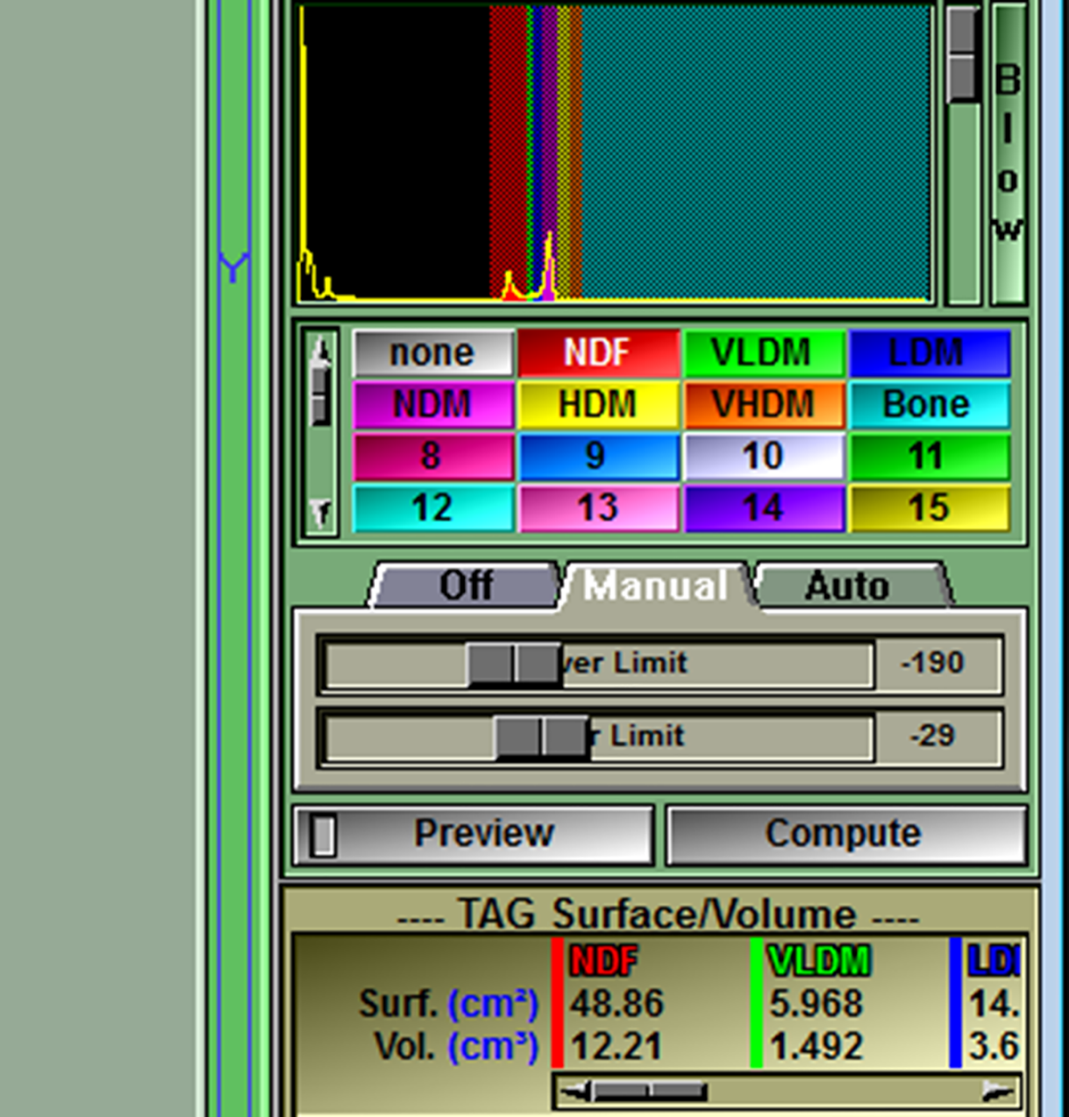
Next click compute in the Control Area to apply the segmented changes to the thigh and show the size of each tagged region (calculated as area under the curve in the histogram). The size for areas of NDF and VLDM may be the only ones seen, but the slider bar allows scrolling to see all areas including Bone at the far right. Also click Preview so that it is turned off or a bug in the program will interfere with the next step.

**Fig 10:**
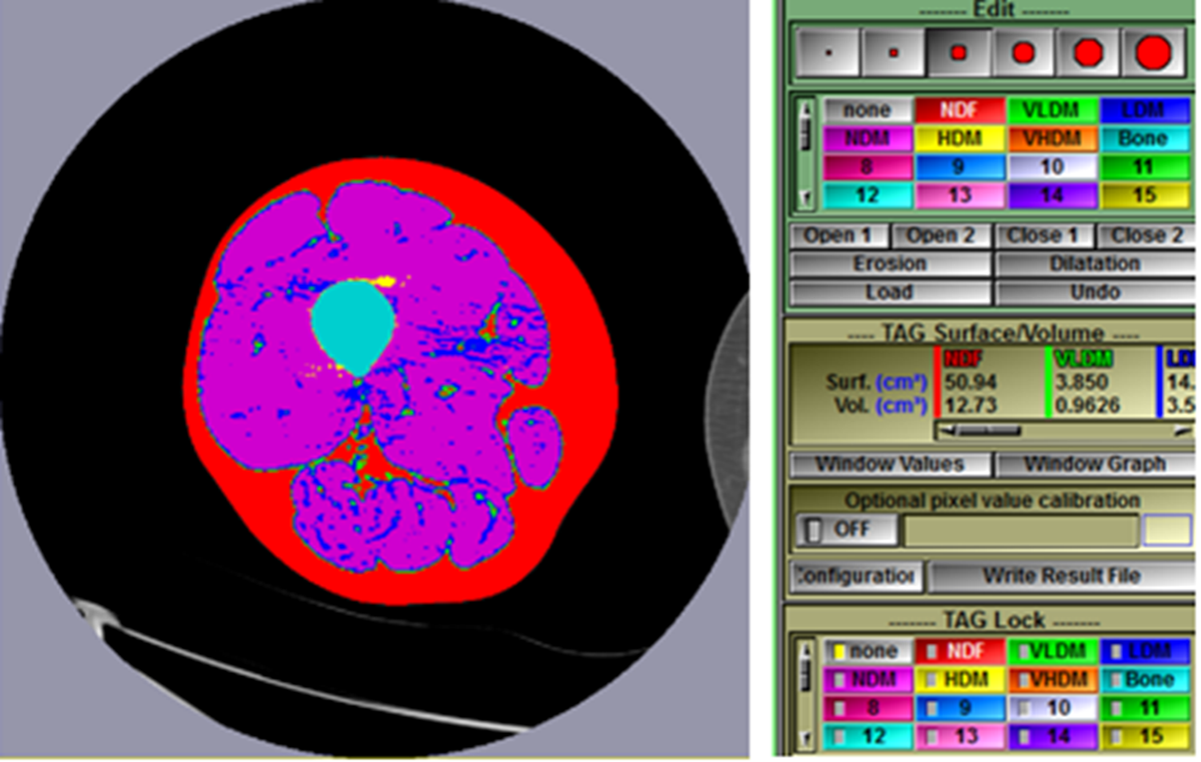
The skin and bone marrow tag with a mixture of colors assigned to the various densities of fat and muscle. Since this could interfere with the results, particularly muscle, we have chosen to edit or brush the skin and structures within the subcutaneous fat (e.g. great saphenous vein) to be normal density fat and brush bone marrow to be bone. From the main menu click on Modes > Edit. A brush size and the desired colored tag must be chosen. The brush is used by holding the left mouse button while dragging the cursor over the area to be edited. This process is easy for marrow since it is surrounded by bone and easy for skin since Tag Lock “none” protects outside the thigh. Brushing can be made easier by increasing the image size in the Display Area by clicking the keyboard plus (+) sign. Similarly, minus (-) decreases the image size. When choosing Edit mode, if the image reverts to the red colored thigh, then either Compute was not selected or Preview was not turned off as instructed in Fig 9. When brushing is complete, if uncolored pixels are present, they can be brushed the desired color after Tag Lock “none” is turned off.

**Fig 11:**
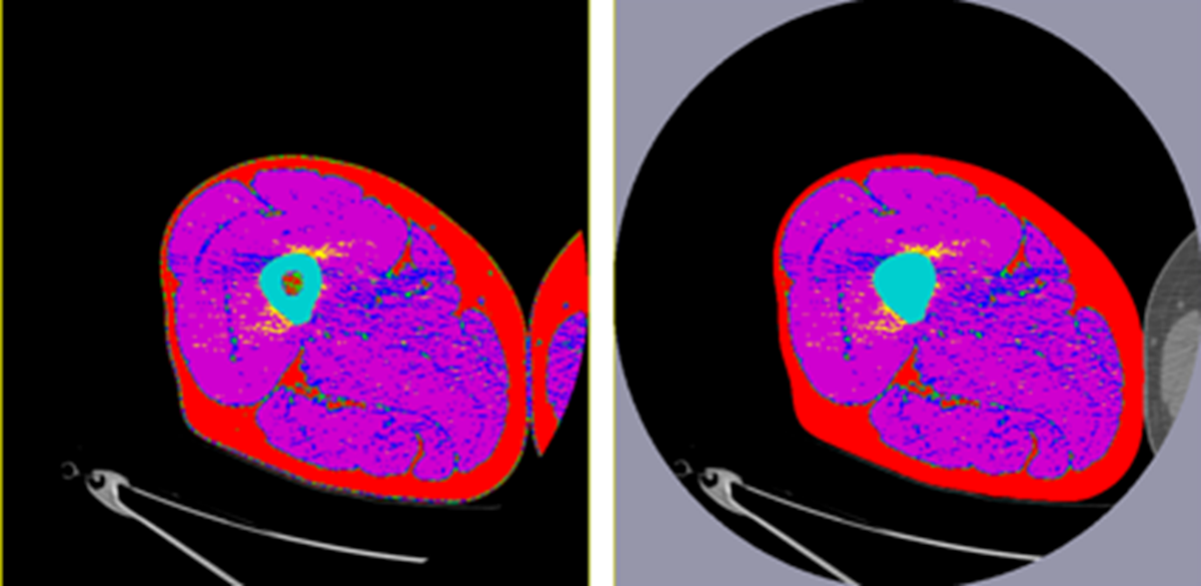
As mentioned in Fig 6, sometimes an attempt to isolate the thigh of interest tags additional areas. Should this happen, it appears easiest to proceed through the instructions in Fig 9. The Editing skills learned in Fig 10 can also be used to limit the tagged areas to the thigh of interest. To edit the touching leg, tag lock “none” will need to be turned off and the inappropriately colored area brushed to the color “none”. The final tagged image can be saved by clicking Main Menu > File > Save TAG Files. The program uses the same filename as the original DICOM file (*.dcm) but adds to the extension (*.dcm.tag) and also saves the TAG file in the same location as the DICOM file. The TAG file will open automatically in the future when the DICOM file is opened. If the original DICOM file needs to be analyzed again, then a copy of the *.dcm file can be saved and analyzed under a new filename.

**Fig 12:**
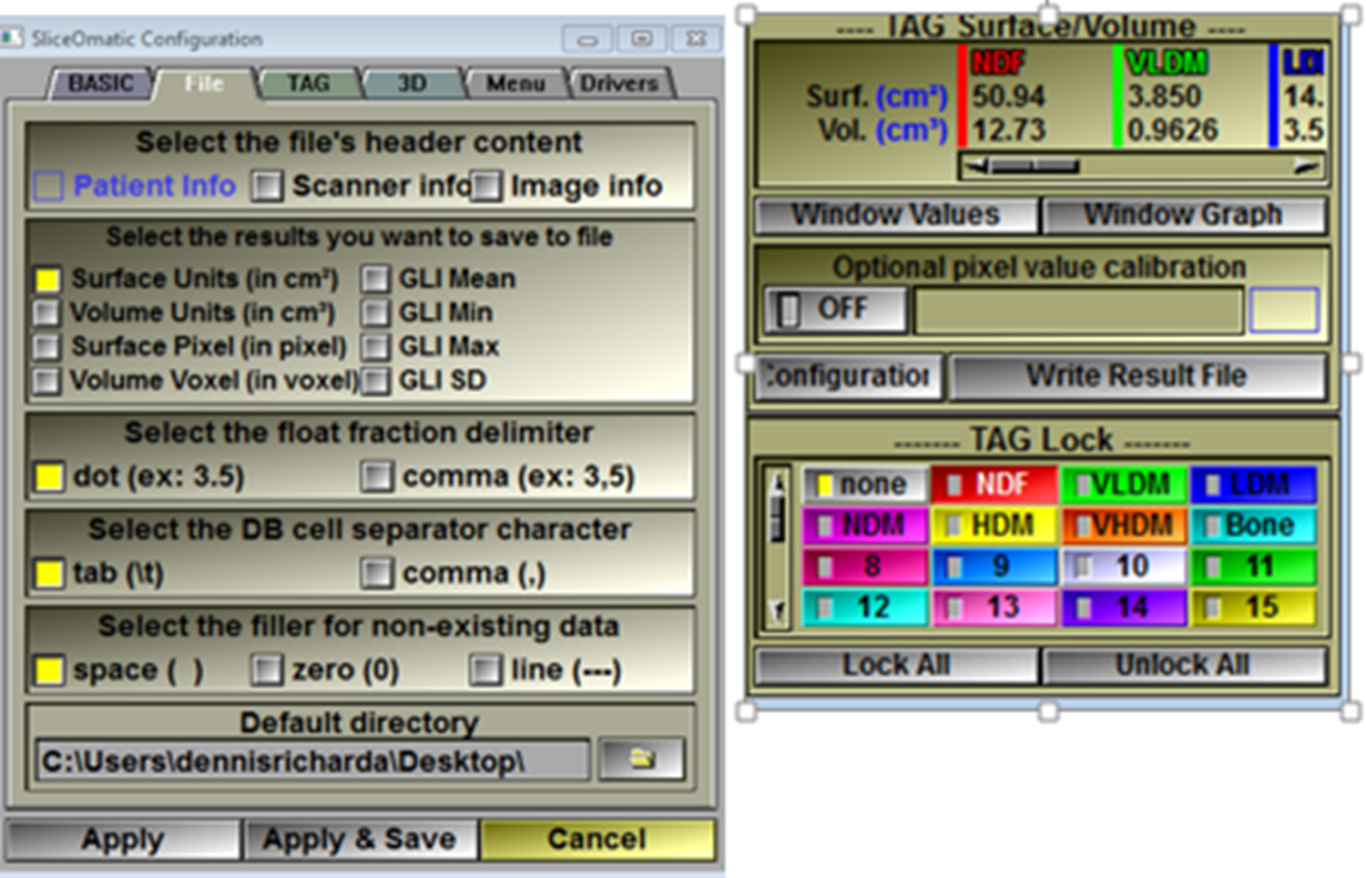
The TAG Surface Areas or results are exported as a CSV file (default name “results.csv”) and the parameters to be exported can be selected. The current analysis was limited to area (cm^2^) as opposed to volume or HU average, minimum, or maximum by choosing Main Menu > File > Config > File > Surface Units (in cm^2^). Unselect any unwanted results and Apply & Save. Export the results by clicking Write Result File in the Control Area.

**Fig 13:**
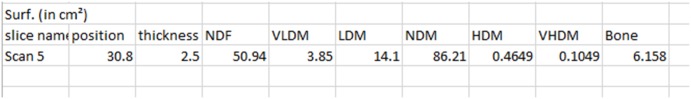
The CSV file (results.csv) can be opened and manipulated using excel and is saved in the same location as the original DICOM file. When additional scans are analyzed and ready to Write Result File, the same result.csv filename will be offered. If chosen, an option will be given to append result.csv by adding the new results to the previous results. The results layout in the CSV file is shown. In addition to the CT scan filename, the columns also contain the scan position and thickness of the scan slice (neither needed for this tutorial), and the surface area (cm^2^) for the regions of interest as abbreviated in Table 1.

CT images were analyzed using commercially available software, SliceOmatic version 5.0 revision 7 (Tomovision, Montreal, Canada) plus its Histogram Segmentation module. The CT image file format was DICOM but the software can read other image formats as well (e.g. TIFF, JPEG). The tissue density ranges used to segment the thigh into tissues is presented in Table 1 and step-by-step instructions for the analysis method are presented in Figs 1–13. It should be noted that the method described here, a histogram segmentation method, cannot be performed exactly as described using prior versions of the software. However, the analyses are possible using prior SliceOmatic versions if an alternate method based on the program’s threshold mode is used. The histogram segmentation tutorial is presented here though both the histogram and threshold segmentation methods are demonstrated in a supplemental youtube.com video [19]. If investigators wish to evaluate the histogram segmentation method and do not have a capable version of the software, then inquiry about obtaining a temporary license can be made by emailing support@tomovision.com. The software was successfully used on computers with Windows 7 and at least an Intel Core Duo CPU E8400 3.00 GHz, 2.0 GB RAM, 64-bit, and ATI Radeon HD 3450 graphics card. SliceOmatic support indicated that the lag time experienced in image refresh rate during this analysis would be resolved by a superior graphics card but this issue did not interfere with the analyses.

**Table 1:** Segmentation of thigh CT images into regions of fat, muscle, and bone based on user-defined Hounsfield unit ranges for tissue density.

Tissue^1^	Tag #	Tag Color^2^	Tag Label^2^	Lower Limit ≥^3^	Upper Limit <^3^	Localization Sites of Tagged Tissues^4^
**Normal Density Fat**	1	Red	Fat	-190	-29	subcutaneous & inter-muscular fat, skin, marrow
**Very Low Density Muscle**	2	Green	VLDM	-29	0	muscle-fat borders, skin, marrow
**Low Density Muscle**	3	Blue	LDM	0	35	inter- and intra-muscular, skin, muscle-fat & marrow borders
**Normal Density Muscle**	4	Pink	NDM	35	101	muscle, skin, marrow
**High Density Muscle**	5	Yellow	HDM	101	151	bone-muscle border, intra-muscular
**Very High Density Muscle**	6	Orange	VHDM	151	200	bone-muscle & bone-marrow borders
**Bone**	7	Light Blue	Bone	200	2001	bone

^1^The areas of intermediate density muscle (VLDM, LDM, HDM, or VHDM) can be considered predominantly muscle based on the effects of averaging pixels for NDF and NDM or NDM and bone when pixels for two tissues overlap in the thickness of the image.

^2^Tissue segmentation is visualized and quantified based on SliceOmatic applying colors or “tags” to tissue regions whose names are abbreviated in the program as a “tag label”.

^3^Tissue density range is presented here as greater than or equal to a lower limit and less than an upper limit to be consistent with their interpretation of program scripts by the SliceOmatic software.

^4^Qualitative assessment of CT scan images (prior to editing) to assess distribution in the thigh of regions tagged by tissue density.

### Statistical analyses

Summary statistics (means ± standard deviations, and ranges) are provided to describe the absolute and relative contributions to total thigh area of regions segmented by tissue density (Objective 2). The effects of image editing to remove interference by skin and marrow on thigh composition were determined by comparing the change in area for each region after editing to zero with a one sample *t*-test. The effects of image editing on the statistical inferences for exercise training were determined by paired sample *t*-test (pre- versus post-training areas) performed separately on data from unedited and edited images (Objective 3). The significance level was 0.05 for all tests and SAS/STAT software, version 9.4, SAS System for Windows (SAS Institute, Cary, NC) was used.

### Supplemental files

Supplemental files are available for download from the journal website to allow SliceOmatic users to follow the tutorial and replicate results. The files are those utilized in the YouTube demonstration. The files include five de-identified scans (filenames Scan 1.dcm to Scan 5.dcm) (S1–S5 Files). The files also include three text files. One text file (Thigh Segmentation Tutorial.scp)(S6 File) is needed to follow the instruction within this manuscript while the other two text files (Thigh Threshold 1.scp and Thigh Threshold 2.scp)(S7 and S8 Files) are needed if the instructions in the YouTube video for using older versions of SliceOmatic are followed. The full dataset (Scan Analysis Dataset.xlsx)(S9 File) for the results presented in this manuscript and the results (Results.csv)(S10 File) for the five practice scans are also available.

## Results

### Subject characteristics

The participants (N = 24) were non-Hispanic males of black (N = 7) or white (N = 17) race. Their mean age was 69 ± 4 yrs (range 62–77 yrs) and BMI was 26.4 ± 2.8 kg/m^2^ (range 20.2–30.7 kg/m^2^). The total area of the mid-thigh for participants before training averaged 220.1 ± 31.6 cm^2^. After training, thigh area increased 8.9 ± 8.4 cm^2^ (P<0.0001) or 4.2 ± 4.2%. The change in thigh size ranged from -4.0 to +11.0%.

### Effect of image editing on thigh regions

All CT images (pre- and post-training) provided by participants were segmented into regions of fat, muscle, and bone defined by Hounsfield unit range for tissue density (Table 1) using the SliceOmatic histogram method (Figs 1–13). The area of each region and their percent contribution to total thigh area were calculated before and after images were edited to account for overlapping tissue density for muscle and fat with skin and bone marrow. Table 2 contains these values and addresses Objective 2. The average thigh size for all scans (pre- and post-training) was 224.5 ± 31.1 cm^2^. Before editing, the largest regions within this area were normal density fat (NDF, 66.2 ± 22.3 cm^2^) and normal density muscle (NDM, 109.0 ± 20.9 cm^2^) which accounted on average for 29.1% and 48.9% of the thigh respectively. In descending order, the contributions by other tissue densities to thigh area were: 4.3% by very low density muscle (VLDM), 14.3% by low density muscle (LDM), 0.79% by high density muscle (HDM), 0.07% by very high density muscle (VHDM), and 2.6% by bone. The sum of all densities of muscle accounted for 68.4% of the thigh. The images were then edited to account for density overlap between skin and marrow with muscle and fat. The areas of NDF (4.2 ± 1.2 cm^2^) and bone (1.4 ± 0.44 cm^2^) increased since skin was edited to be NDF and marrow to be bone (P<0.0001 each). The other areas of the thigh decreased as expected since contributions to their area by skin and marrow were removed (P<0.0001 each). Editing changed the percent contribution of each region to total area by less than two percent on average but there was some variability between individuals as shown by the range of contributions for each region (Table 2). The sum of all muscle densities changed from 68.4% to 65.8% of the thigh after editing. The reliability of these assessments was also determined. Between an experienced and a novice user of the software, the R^2^ for values collected was 1.0, and for duplicate ratings by a novice user, the coefficient of variation was 0.06% +/- 0.28.

**Table 2:** Effect of CT image editing of skin and bone marrow on contributions of muscle, fat, and bone to total thigh area.

Tissue	Before Editing			After Editing			
	Area^1^^,^^2^ (cm^2^)	% of Total Thigh^2^	% of Total Thigh^3^	Change in Area (cm^2^)	P-Value^4^	% of Total Thigh^2^	% of Total Thigh^3^
^5^**Normal Density Fat (NDF)**	66.2 ± 22.3	29.1 ± 7.4	14.7–42.0	4.2 ± 1.2	<0.0001	30.9 ± 7.4	17.0–43.2
^5^**Very Low Density Muscle (VLDM)**	9.6 ± 2.6	4.3 ± 1.0	2.6–7.1	-2.6 ± 0.48	<0.0001	3.2 ± 1.1	1.3–6.0
^5^**Low Density Muscle (LDM)**	31.9 ± 8.7	14.3 ± 3.9	6.2–22.4	-2.2 ± 0.69	<0.0001	13.3 ± 3.8	5.0–21.3
^5^**Normal Density Muscle (NDM)**	109.0 ± 20.9	48.9 ± 8.2	33.5–62.7	-0.72 ± 0.50	<0.0001	48.5 ± 8.2	33.3–62.6
^5^**High Density Muscle (HDM)**	1.9 ± 1.3	0.79 ± 0.50	0.18–2.0	-0.06 ± 0.04	<0.0001	0.77 ± 0.50	0.16–2.0
^5^**Very High Density Muscle (VHDM)**	0.17 ± 0.06	0.07 ± 0.02	0.04–0.14	-0.05 ± 0.03	<0.0001	0.05 ± 0.02	0.03–0.12
^5^**Bone**	5.7 ± 0.5	2.6 ± 0.36	2.0–3.4	1.4 ± 0.44	<0.0001	3.2 ± 0.47	2.5–4.6

^1^All pre- and post-training scans (N = 48) were included in the data. Average area of all thigh scans was 224.5 ± 31.1 cm^2^.

^2^Data shown as mean ± SD.

^3^Data shown as range.

^4^Signficance of absolute change in area due to editing of the thigh region.

^5^Hounsfield unit range for each segmented region: NDF (-190 to -30), VLDM (-29 to -1), LDM (0 to 34), NDM (35 to 100), HDM (101 to 150), VHDM (151 to 199) and Bone (200 to 2000).

### Effect of image editing on training results

Thigh tissue areas pre-training and their post-training changes in size are presented to address Objective 3 (Table 3). Before image editing, training resulted in significant increases in muscle for VLDM (5.5 ± 7.9%, P = 0.003), LDM (9.6 ± 16.8%, P = 0.01), NDM (4.2 ± 7.9%, P = 0.02), and HDM (70.9 ± 80.6%, P = 0.0004). NDF, VHDM, and bone did not change (P>0.05 each). The magnitude of these changes after training varied greatly between individuals as indicated by the standard deviations that are equal to or greater than the means. This variability is also apparent from the negative lower limit for range of change indicating that some participants had decreases in area for muscle and fat. For example, the change in normal density muscle ranged from -10.6 to +18.8%. These results were not affected by editing the images to account for skin and bone marrow. Significant changes after training remained significant and insignificance remained insignificant regardless of editing (Table 3). This lack of an effect of editing was confirmed for each region by comparing the post-training changes before and after editing and none were significantly different (P>0.05 each, Table 3).

**Table 3:** Effect of image editing on cross-sectional areas of thigh tissues and their change after 12-wks of resistance training (N = 24).

	Before Editing^1^				After Editing^1^			
	Pre-Training^2^ (cm^2^)	Post-Training Change^2^^,^^3^ (cm^2^)	Relative Change^4^ (%)	P-Value	Pre-Training^2^ (cm^2^)	Post-Training Change^2^^,^^3^ (cm^2^)	Relative Change^4^ (%)	P-Value
^5^**Normal Density Fat (NDF)**	65.9 ± 23.0	0.60 ± 5.4	1.8 ± 8.8 (-14.3–24.0)	0.5940	70.0 ± 23.5	0.74 ± 6.0	2.1 ± 9.3 (-15.5–27.1)	0.5483
^5^**Very Low Density Muscle (VLDM)**	9.4 ± 2.4	0.53 ± 0.76	5.5 ± 7.9 (-11.7–20.7)	**0.0025**	6.8 ± 2.3	0.66 ± 1.0	9.4 ± 14.0 (-16.0–47.7)	**0.0045**
^5^**Low Density Muscle (LDM)**	30.7 ± 8.4	2.5 ± 4.6	9.6 ± 16.8 (-23.9–46.0)	**0.0123**	28.6 ± 8.2	2.40 ± 4.6	9.8 ± 17.3 (-22.2–44.1)	**0.0168**
^5^**Normal Density Muscle (NDM)**	106.9 ± 20.4	4.3 ± 8.7	4.2 ± 7.9 (-10.6–18.8)	**0.0244**	106.2 ± 20.4	4.12 ± 8.9	4.1 ± 8.1 (-10.7–18.3)	**0.0332**
^5^**High Density Muscle (HDM)**	1.4 ± 0.84	0.93 ± 1.1	70.9 ± 80.6 (-49.5–327.2)	**0.0004**	1.3 ± 0.84	0.94 ± 1.1	78.4 ± 90.2 (-50.0–360.9)	**0.0004**
^5^**Very High Density Muscle (VHDM)**	0.17 ± 0.06	-0.003 ± 0.04	1.1 ± 22.1 (-36.7–69.8)	0.7157	0.12 ± 0.05	-0.001 ± 0.03	2.6 ± 27.3 (-33.3–77.1)	0.9013
^5^**Bone**	5.7 ± 0.46	-0.002 ± 0.08	-0.03 ± 1.4 (-3.5–2.9)	0.8958	7.1 ± 0.70	-0.0005 ± 0.05	-0.01 ± 0.71 (-2.0–1.0)	0.9639

^1^Due to overlap in density with segmented regions of muscle and fat, the skin and blood vessels within subcutaneous normal density fat were edited to be normal density fat (NDF) and bone marrow was edited to be bone.

^2^Area data in shown as Mean ± SD.

^3^Post-training changes were compared before and after editing and were not significantly different for any tissue region (P>0.05 each).

^4^Percent change from pre- to post-training data shown as Mean ± SD and (range) and P-values for the change are presented.

^5^Hounsfield unit range for each segmented region: NDF (-190 to -30), VLDM (-29 to -1), LDM (0 to 34), NDM (35 to 100), HDM (101 to 150), VHDM (151 to 199) and Bone (200 to 2000).

## Discussion

A quick start method has been provided for using SliceOmatic to calculate areas of muscle, fat, and bone from CT images of the thigh. This work was motivated by the serious impact that aging has on muscle health and our need for a consistent reliable method to determine muscle size and composition [11, 12]. The method showed an inter-user reliability of R^2^ = 1.0 and intra-user reliability of CV 0.1%. Equally reliable results can be generated using other software such as NIH Image J for the same purpose. However, analysis with Image J does not easily account for density overlap between muscle and fat versus skin which contains a substantial amount of tissue defined here as VLDM (-29 to 0 HU). Thus, the current work sought to determine if editing skin to be subcutaneous fat using this SliceOmatic method matters or if contributions of skin to muscle and fat can be left uncorrected. This is an important issue since editing the thigh images to account for tissue density overlap could be important technically and/or physiologically.

Thigh composition of our participants (62–77 yrs, BMI 20–31) averaged 49% NDM and 29% NDF. LDM made up 14% of the thigh while other densities contributed less (VLDM 4%, bone 3%, HDM 1%, VHDM 0.1%). These values were minimally (<2%) affected by density overlap with skin and marrow. Furthermore, our participants experienced a 4% increase in thigh size after resistance training due to gains in tissues from -29 to +150 HU. These results also were negligibly affected by image editing. Thus, accounting for density overlap of skin and marrow with muscle and fat can easily be performed with SliceOmatic when technical precision is preferred; however, density overlap did not alter our findings for resistance training and editing is apparently not essential if only Image J is available.

To our knowledge, this is the first presentation of thigh CT analyses that has included all densities from -190 to +2000 HU even though our results (VLDM 4% + 1% HDM) and others indicate that omission of any density range could exclude a significant amount of tissue [5]. The range -190 to -30 HU is consistently considered normal density fat [5, 20]. We considered labeling -29 to -1 HU as high density fat due to its density less than water. However, others consider the area muscle since it localizes to muscle-fat borders and is due to density averaging when pixels contain both muscle and fat [8, 21]. Our results support this approach and labeling the -29 to -1 HU area as VLDM since it increased with exercise training. The range 0 to +34 HU for LDM is often considered separately due to it having higher lipid content than NDM in the +35 to +100 HU range [10, 22]. The range +101 to +150 HU or HDM was small (1%) and though it mostly appeared around the femur, pixels were sometimes scattered throughout the thigh muscles. Thus, it remains to be determined if this is normal muscle or if there is a macromolecular basis, like low density muscle, for its altered density. The range +151 to +199 HU localized to a few (0.1%) pixels at the muscle-bone border and can be considered muscle even though the contribution is small (0.1%). And finally, pixels with a density of over +200 HU localized solely to bone.

### Conclusions

A simple tutorial has been presented for using SliceOmatic to quantify areas of muscle, fat, and bone in CT images of the thigh. The method was developed using a cohort of all males due to the demographics Veterans, aged 60–80, but this does not interfere with application of the method to other aged populations [23]. The tutorial was not comprehensive in that alternate methods can be used and the collection of density data based on mean attenuation was not covered. However, two important methodological issues were examined. Accounting for tissue density overlap with skin and marrow may not be necessary since they had minimal effect on the contributions of muscle and fat to total thigh and did not affect the statistical inferences of exercise training. When using SliceOmatic to calculate thigh composition, we recommend examining tissue density from -190 to +2000 HU so that the total thigh is included. Muscle analysis can be split into multiple densities from -29 to +200 HU in case a certain range (e.g. LDM 0 to +34 HU) is related to the health condition and intervention of interest. However, if distinct relationships with smaller ranges are not found, then we recommend that total muscle is defined as -29 to +200 HU.

## Supporting information

S1 File This is the Scan 1.dcm file. This is the CT image 1 to allow user to allow user to practice the tutorial.
(DCM)

S2 File This is the Scan 2.dcm file. This is the CT image 2 to allow user to allow user to practice the tutorial.
(DCM)

S3 File This is the Scan 3.dcm file. This is the CT image 3 to allow user to allow user to practice the tutorial.
(DCM)

S4 File This is the Scan 4.dcm file. This is the CT image 4 to allow user to allow user to practice the tutorial.
(DCM)

S5 File This is the Scan 5.dcm file. This is the CT image 5 to allow user to allow user to practice the tutorial.
(DCM)

S6 File This is the Thigh Segmentation Tutorial.scp file. This is the script file necessary to replicate the method.
(SCP)

S7 File This is the Thigh Threshold 1.scp file. This is the script file 1 for the alternate method presented on YouTube.
(SCP)

S8 File This is the Thigh Threshold 2.scp file. This is the script file 2 for the alternate method presented on YouTube.
(SCP)

S9 File This is the Scan Analysis Dataset.xlsx file. This is the data file for the results presented in this manuscript.
(XLSX)

S10 File This is the Results.csv file. This is the data file for results of the five practice scans demonstrated on YouTube.
(CSV)
